# Analysis of work ability and work-related physical activity of employees in a medium-sized business

**DOI:** 10.1186/s13104-015-1781-9

**Published:** 2015-12-18

**Authors:** Christiane Wilke, Philip Ashton, Tobias Elis, Bianca Biallas, Ingo Froböse

**Affiliations:** Institute for Movement Therapy and Movement-Oriented Prevention and Rehabilitation, German Sports University Cologne, Am Sportpark Müngersdorf 6, 50933 Cologne, Germany

**Keywords:** Workplace health promotion, Blue-collar worker, White-collar worker, Activity level, Ageing workforce

## Abstract

**Background:**

Work-related physical activity (PA) and work ability are of growing importance in modern working society. There is evidence for age- and job-related differences regarding PA and work ability. This study analyses work ability and work-related PA of employees in a medium-sized business regarding age and occupation.

**Methods:**

The total sample consists of 148 employees (116 men—78.38 % of the sample—and 32 women, accounting for 21.62 %; mean age: 40.85 ± 10.07 years). 100 subjects (67.57 %) are white-collar workers (WC), and 48 (32.43 %) are blue-collar workers (BC). Work ability is measured using the work ability index, and physical activity is obtained via the Global Physical Activity Questionnaire.

**Results:**

Work ability shows significant differences regarding occupation (p = 0.001) but not regarding age. Further, significant differences are found for work-related PA concerning occupation (p < 0.0001), but again not for age. Overall, more than half of all subjects meet the current guidelines for physical activity.

**Conclusion:**

Work ability is rated as good, yet, a special focus should lie on the promotion during early and late working life. Also, there is still a lack of evidence on the level of work-related PA. Considering work-related PA could add to meeting current activity recommendations.

## Background

Demographic ageing is a major issue in today’s society. This is due to a decreasing birth rate and mortality rate [1]. While less children are being born in Germany, more individuals reach an increasing age. According to the European Commission [2] this has three specific impacts for Germany. First, the population will decrease from about 83 million in 2010 to 75 million in 2050. Second, life expectancy will increase from 77 years (men) and 82 years (women) to 83 and 88 respectively. Third, below-replacement fertility will lead to a mushroom-cloud shaped age structure. This demographic change affects the German economy as one out of four employees (27 %) is 50 years and older, yet only one in five is below the age of 30 [3]. This has further implications for German businesses. On the one hand employers are hit by talent shortages and on the other hand they need to face the challenge to bind elder employees until retirement. Thus, employers need to invest in the promotion of both younger and elder employees [4]. Minimising the impact of demographic change solely is insufficient. Hence, demographic change is to be considered as a future challenge in the context of politics, economy and research.

Work ability is defined as a person’s capability of coping with a given task at a given point of time [5]. Work ability influences an employee’s well-being and decreases the risk of musculoskeletal diseases, sick leave and early retirement. Further, a low work ability affects the company’s productivity [6] and, thus, its ability to compete on the market. Therefore, work ability is to be seen as an interaction between the employee’s individual resources (e.g. health status) and the workplace condition. Studies have shown that the kind of occupation takes an impact on a person’s work ability. For instance, white-collar workers exhibit higher levels of work ability than blue-collar workers [5]. Also, the probability of a low work ability increases with age, yet it can be promoted with advanced age [7]. Factors influencing one’s work ability increasingly start to become apparent in middle age [6]. Hence, work ability should not be promoted among all employees equally, but should also focus on the phase of early and late working life in order to ensure work ability until retirement.

With technology and industrial automation still advancing rates of physical activity and metabolism have been decreasing in working society for the last decades. Considering physical activity (PA) at the workplace, the exclusion of work-related PA often leads to a significant underestimation of his overall PA by the employee [8]. Thus, work-related PA should be examined separately from overall PA. Work-related PA has increased by over 5 % since the 1990s [9], especially with men [10]. Furthermore, work-related PA shows a moderate level of overall inactivity with people between 60 and 65 years showing the highest level [11]. However, the percentage of moderate-intensive activities at work decreased between 1960 and 2010 by nearly 28 %, yet low-intensive and sedentary activities rose by 17 and 9 % respectively within the same period. Blue-collar workers show higher rates of work-related PA than white-collar workers, hence, their leisure-time serves as a measure of regeneration rather than further physical demands [12]. In conclusion, considering work-related PA could add to meeting current activity recommendations [8]. Therefore, this study analyses work ability and work-related PA of employees in a medium-sized business regarding age and occupation.

The present study analyses both work ability and PA in the same context, as the level of PA influences a person’s health status and performance and, thus, could influence his work ability.

## Methods

### Sample

The study sample comprises white-collar (WC) and blue-collar (BC) workers of a medium-sized business of the chemical industry. Within the present study commercial staff members are defined as white-collar workers. Industrial employees belong to the group of blue collar workers. The study was conducted in Germany. All subjects tested are seen as a positive selection of the company’s workforce as they took part voluntarily showing a general interest in health-related topics. All of the company’s employees were invited to take part in the present study. The total participation rate of 26.1 % is rather low compared to other studies (30–50 %) [13, 14]. A further n = 3 participants were withdrawn from the analysis due to missing values leading to a final sample size of 148 (98.9 %). The total group of 148 employees comprises more male than female subjects and more WC than BC respectively. Referring to the German Federal Statistical Office, all subjects are assigned to either of the three age groups [15] (see Table 1).

**Table 1:** Demographic data of the sample group

Variable	Frequency (%)
Sex	
Male	116 (78.38 %)
Female	32 (21.62 %)
Total	148 (100 %)
Age (years)	
x ± s	40.85 ± 10.07
18–29 (AG1)	26 (17.57 %)
30–49 (AG2)	91 (61.49 %)
50–65 (AG3)	31 (20.95 %)
Total	148 (100 %)
Occupation	
WC	100 (67.57 %)
BC	48 (32.43 %)
Total	148 (100 %)

*x* mean, *s* standard deviation

### Instruments

The employees work ability is analysed using the Work Ability Index [7] (WAI). The questionnaire evaluates a person’s work ability and defines goals of possible measures. Based on the subject’s individual condition, it describes to what extent he or she is able to carry out his work [6]. The short form of the WAI is used for this study consisting of 10 Items and 7 dimensions. The work ability is evaluated by summing up the score points of every dimension with a total score between 7 and 49. The WAI shows a good reliability in international versions as well as the German language version [16–18].

Data on the work-related PA is collected using the Global Physical Activity Questionnaire (GPAQ) which was developed by the World Health Organisation [19]. This questionnaire consists of 16 Items and 4 dimensions and measures a person’s PA regarding work, transport behaviour and leisure time via individual perception and through behaviour-specific questions. The work dimension is only used in this study. Physical activity is divided into moderate-intensive and vigorous-intensive activities, each performed continuously for a minimum of 10 min. Based on this, the subject’s individual activity level is calculated. The GPAQ’s reliability is moderate [20].

Ethical approval was obtained by the ethics commission of the German Sport University Cologne. Written informed consent was obtained from all participants prior to begin of the study.

### Data collection

All data was collected within the scope of the work place health management. The questionnaires are handed out in paper and pencil format to WC in their office and to BC in groups of 10 employees. A total of 20 min are planned for each employee for answering the questions. The actual response time is estimated with 15 min, thus, an additional 5 min are ensured in case of further questions by the employee. Based on these data all employees are assigned to either of the job groups as well as age groups.

### Analysis

The statistical analysis is carried out with SPSS 21.0 (Statistical Package for the Social Sciences). Differences between the job groups are determined with the Chi squared test and the *t* test for independent samples. Differences between the three age groups are tested using the Chi squared test and ANOVA.

## Results

Regarding occupation WC show higher levels of work ability than BC. No significant results are found for age-related differences in work ability (see Table 2).

**Table 2:** Differences in work ability regarding occupation and age (n = 148)

	N	Score points (x ± s)	P-value (2-sided)
WAI			
WC	100	43 ± 3.93	0.001**
BC	48	40 ± 4.14	
AG1	26	43 ± 4.51	0.225
AG2	91	42 ± 3.93	
AG3	31	41 ± 4.38	

*x* mean, *s* standard deviation; ** p ≤ 0.001

The great part of all subjects shows a low level of PA, yet over half of all employees meet the current guidelines of 2.5 h of physical activity. However, WC are less able to meet the guidelines than BC. The Chi squared test shows a significant difference. Employees from AG2 meet the activity guidelines more frequently than employees from AG1 or AG3, yet there is no significant difference (see Table 3).

**Table 3:** Level of physical activity of all subjects as well as separated for age and occupation (n = 148)

	Level of physical activity						P-value (2-sided)
	Low		Moderate		High		
	N	%	N	%	N	%	
Total	63	42.86	36	24.49	48	32.65	0.514
WC	51	51.52	27	27.27	21	21.21	0.000***
BC	12	25.00	9	18.75	27	56.25	
AG1	13	50.00	3	11.54	10	38.46	0.618
AG2	35	38.89	25	27.78	30	33.33	
AG3	15	48.38	8	25.81	8	25.81	

*** p < 0.001

Regarding work-related PA the results reveal that BC reach a higher level than WC (96.41 ± 143,90 MET-min; 3.02 ± 26.02 MET-min; p < 0.0001). No significant differences are found concerning age (p = 0.541; see Table 4).

**Table 4:** Work-related PA (MET-min) regarding occupation and age (n = 148)

	N	MET-minutes per day (x ± s)	P-value (2-sided)
Pworkday			
WC	100	3.02 ± 26.02	0.000***
BC	47	96.41 ± 143.90	
AG1	26	22.50 ± 79.91	0.541
AG2	90	39.77 ± 106.11	
AG3	31	21.57 ± 64.71	

*x* mean, *s* standard deviation; *** p < 0.001

## Discussion

Regarding occupation the results show a significant difference in work ability between WC and BC. Other research indicates that work ability varies between occupations [21, 22]. The authors postulate that the higher the percentage of (heavy) physical loading during work is the poorer is the level of work ability. However, the level of work ability of BC is good (see Table 2). Few studies have confirmed this statement so far [23]. A possible explanation can be found in the automation of the production chain and its effect on the job requirements by reducing heavy loads to a minimum.

Nearly half of all subjects merely show a low level of total physical activity and, consequently, do not meet the activity guidelines of 2.5 h of physical activity per week set up by the American College of Sport Medicine [24]. In contrast, over half of the subjects meet the activity guidelines by reaching a moderate or high level of total physical activity. This finding does not comply with other research indicating that approximately 60 % of all German adults do not meet the physical activity guidelines, and that only 40 % are physically active for at least 2.5 h per week [25]. The ambivalence between the studies might be due to the different use of subjective data and their bias. The comparison of the age groups shows that AG1 and AG3 hold a similar percentage of low active employees (see Table 3). Hence, nearly one out of two employees, young and old, is not physically active enough. Further, AG3 reveals the lowest percentage of high active employees, with only one out of four elderly workers meeting the activity guidelines. However, in case of AG1 and AG2 this percentage is 38.5 and 33.3 % respectively. This matches other findings [26, 27]. As a conclusion, elder employees are not necessarily less active, yet the present study underlines the evidence so far that elder employees have a higher probability of not meeting current activity guidelines [26]. Regarding occupation the results indicate that nearly half of all WC and 75.0 % of all BC meet the activity guidelines. It seems as if BC are less likely to be low active, yet more likely to be high active and to meet the guidelines than WC. Other studies also show an inconsistency on this matter, therefore, further research needs to be conducted [28].

No differences are found among the three age groups in respect to work-related PA (see Table 4). This is mainly due to the high standard deviation and the fact that younger and older employees reveal the same work-related PA. Current research still shows an ambivalence on this matter. On the one hand, studies conclude that PA during work declines with increasing age [29]. On the other hand, studies underline that the percentage of a sedentary job and low physical activity declines with increasing age [12, 30]. Further research should focus on the significance of work-related PA for elder employees. Comparing both occupations the results indicate a significant difference. WC are as good as physical inactive during their working hours. Physical inactivity is one of the reasons for the high percentage of WC with a low activity rate (see Table 3) and, thus, needs to be discussed in terms of a misjudgement by the employee. While other studies state that WC show a mean activity rate of 700 MET-minutes [8, 31, 32], WC in the present study show a mean activity rate of merely 100 MET-Minutes (see Table 4). The standard deviation poses a high disparity which can be argued as a high interindividuality within this occupation. Other studies underline these findings [8, 31]. Further explanations lie in the diverse use of tools for measuring physical activity as well as in differences regarding social economic status in research so far. It is recommended that further studies validate the existing subjective data of physical activity with new objective measures in order to ensure a higher transparency.

The results show no significant difference concerning work ability (see Table 2). All the three age groups reveal a good level of work ability. Other studies, however, underline that age does not necessarily affect work ability but that the probability of low work ability is greater with increasing age [33, 34]. Other research indicates that even if the variance increases interindividually with age, it still can be observed at younger ages [6]. Considering the standard deviation, it becomes apparent that the variance rather inclines with increasing age (see Table 2). Yet, a growing number of studies conclude that no age-related difference can be found regarding work ability [35]. In a study by WILKE and colleagues [36] the effect of workplace health promotion on work ability was analysed regarding younger (<45 years) and older employees (≥45 years). The authors conclude that no age-related difference exists.

Despite the fact that the percentage of male workers is higher in manufacturing companies, this finding stands against the general knowledge that women in particular show greater interest in health-relevant topics [37]. Due to this disparity no conclusions can be made with regard to sex. The mean age is regarded as average and is in line with comparative studies [14, 36]. Studies have also shown that WC take part in surveys more regularly than BC [38]. The present study gives further evidence on this topic. Socially desirable responding is an important issue when conducting surveys. Chances are that employees tend to present themselves in a positive way and, thus, do not respond correctly and honestly. The growing importance of the employee’s performance in today’s society may be a reason for that, yet it needs to be considered when drawing conclusions from the present results.

## Conclusion

This study provides further evidence on work ability and physical activity, especially in relation to occupation and age. While the results regarding work ability and age-related physical activity are in line with existing findings, the results on physical activity regarding occupation are inconsistent. Therefore, further evidence is needed on this topic. The role of work-related PA is yet to be recognised in both research and society. Promoting physical activity on the company’s premises is inevitable. The implementation of corresponding measures lies in the interest of employer, employee and society.
